# Combustion-derived flame generated ultrafine soot generates reactive oxygen species and activates Nrf2 antioxidants differently in neonatal and adult rat lungs

**DOI:** 10.1186/1743-8977-10-34

**Published:** 2013-08-01

**Authors:** Jackie KW Chan, Jessica G Charrier, Sean D Kodani, Christoph F Vogel, Sarah Y Kado, Donald S Anderson, Cort Anastasio, Laura S Van Winkle

**Affiliations:** 1 Center for Health and the Environment, University of California, One Shields Ave, Davis, CA 95616, USA; 2 Land Air and Water Resources (LAWR), University of California, Davis One Shields Ave, Davis, CA 95616, USA; 3 Department of Environmental Toxicology, University of California, Davis One Shields Ave, Davis, CA 95616, USA; 4 Department of Veterinary Medicine: Anatomy, Physiology and Cell Biology, University of California, Davis One Shields Ave, Davis CA95616-8732, USA

**Keywords:** HOOH, Hydrogen peroxide, Hydroxyl radical, Lung development, OH, O2, Reactive oxygen species, ROS, Superoxide

## Abstract

**Background:**

Urban particulate matter (PM) has been epidemiologically correlated with multiple cardiopulmonary morbidities and mortalities, in sensitive populations. Children exposed to PM are more likely to develop respiratory infections and asthma. Although PM originates from natural and anthropogenic sources, vehicle exhaust rich in polycyclic aromatic hydrocarbons (PAH) can be a dominant contributor to the PM_2.5_ and PM_0.1_ fractions and has been implicated in the generation of reactive oxygen species (ROS).

**Objectives:**

Current studies of ambient PM are confounded by the variable nature of PM, so we utilized a previously characterized ethylene-combusted premixed flame particles (PFP) with consistent and reproducible physiochemical properties and 1) measured the oxidative potential of PFP compared to ambient PM, 2) determined the ability of PFPs to generate oxidative stress and activate the transcription factor using *in vitro* and *ex vivo* models, and 3) we correlated these responses with antioxidant enzyme expression *in vivo*.

**Methods:**

We compared oxidative stress response (HMOX1) and antioxidant enzyme (SOD1, SOD2, CAT, and PRDX6) expression *in vivo* by performing a time-course study in 7-day old neonatal and young adult rats exposed to a single 6-hour exposure to 22.4 μg/m^3^ PFPs.

**Results:**

We showed that PFP is a potent ROS generator that induces oxidative stress and activates Nrf2. Induction of the oxidative stress responsive enzyme HMOX1 *in vitro* was mediated through Nrf2 activation and was variably upregulated in both ages. Furthermore, antioxidant enzyme expression had age and lung compartment variations post exposure. Of particular interest was SOD1, which had mRNA and protein upregulation in adult parenchyma, but lacked a similar response in neonates.

**Conclusions:**

We conclude that PFPs are effective ROS generators, comparable to urban ambient PM_2.5,_ that induce oxidative stress in neonatal and adult rat lungs. PFPs upregulate a select set of antioxidant enzymes in young adult animals, that are unaffected in neonates. We conclude that the inability of neonatal animals to upregulate the antioxidant response may, in part, explain enhanced their susceptibility to ultrafine particles, such as PFP.

## Introduction

Urban particulate matter (PM) exposure has been epidemiologically correlated with multiple cardiopulmonary morbidities and mortalities, especially in susceptible populations [1-4]. Of special concern are the smaller fractions of PM: fine PM_2.5_ particles with an aerodynamic diameter (AD) of less than 2.5 μm, and ultrafine PM_0.1_ particles with AD of less than 0.1 μm. These fractions can penetrate deep into the bronchiolar and alveolar regions of the lung [5]. Although particulate pollution originates from both natural and anthropogenic sources, vehicle exhaust can be a dominant contributor of the PM_2.5_ and PM_0.1_ fractions [6]. Vehicle exhaust from combustion of gasoline, diesel and other petroleum fuels contains carbonaceous particles with fused and free polycyclic aromatic hydrocarbons (PAHs). It has been estimated that vehicle exhaust contributes to over 50% of urban PM_2.5_ mass [7].

Children are a susceptible population to inhaled PM. Several physiological factors play a role in enhanced susceptibility. Compared to adults, children are more aerobically active outdoors, have a larger body surface area-to-volume ratio, higher metabolic rate, and have increased minute ventilation and oxygen consumption [8]. Moreover, the lung continues to mature, develop and grow postnatally. The developing lung undergoes continuous alveolarization, cellular maturation and differentiation that occurs up to the first 8 years of a child’s life [9,10]. This may be disrupted by exposure to air pollution during these critical years. Epidemiologic studies have shown that children who live near roadways with high levels of vehicle traffic are more predisposed to both the development and exacerbation of asthma, have a higher incidence of pneumonia and bronchitis and are more likely to miss school as a result [11]. PM_0.1_ exposure has also been linked to diminished lung development and reduced lung function in children [12].

Reactive oxygen species (ROS) generated from PM have been implicated in the generation of cellular oxidative stress in both *in vivo* and *in vitro* models [13,14]. Oxidative stress occurs upon a loss of cellular homeostasis, where ROS generation overwhelms antioxidant defenses. This can directly result in cytotoxicity [15] and the activation of specific transcriptional pathways, such as Nrf2, that are responsible for upregulating phase II antioxidant gene expression [16]. The lung is rich in non-enzymatic antioxidants, such as reduced glutathione, ascorbic acid (Vitamin C), α-tocopherol (Vitamin E), lycopene, and β-carotene [17]. Further, systems of persistent and inducible antioxidant enzymes are uniquely equipped to reduce intracellular ROS [18,19], restoring cellular homeostasis.

Many toxicological studies use field-collected ambient PM as the exposure regimen. Results from these studies are confounded by the variable nature of ambient PM. The composition of ambient PM is dependent on the time of day, season, weather and location. This complicates the systematic attribution of health effects to a single component or group of components and makes exact reproduction of such studies challenging. To address this issue, we have developed and characterized a system to generate premixed flame particle (PFP) [20] that are rich in PAHs, especially naphthalenes, to simulate the effects of inhaling vehicle exhaust. We have previously shown that animals exposed to PFPs have increased cellular toxicity. Further, following exposure there are age-dependent and lung compartmental changes in levels of non-enzymatic (GSH, GSSG) and enzymatic (GCL, GSR, GPX, GSTs) antioxidants [21-23].

Building upon our previous work, the current study was designed to address three goals: (1) to determine whether PFPs generate ROS, (2) to examine whether changes in enzymatic antioxidant expression *in vivo* are correlated with ROS generation, and (3), to examine age-specific antioxidant expression in response to PFP exposure. We hypothesized that PFPs generate ROS, which causes lung oxidative stress, and that the inability of neonates to upregulate antioxidant enzymes in response to elevated oxidative stress results in enhanced epithelial susceptibility. To test our hypothesis, we divided the current study into two parts. First, we analyzed the oxidative potential of PFPs by measuring ROS generation in a surrogate lung fluid (SLF), and reinforced these results using *in vitro* and *ex vivo* treatment models. In the second part, we performed a time-course study where 7-day old neonatal pups and 8-week old young adult rats were whole-body exposed to an acute 6-hour exposure of PFPs, and we then compared antioxidant enzyme expression that can functionally reduce the measured ROS.

## Results

### PFPs generate reactive oxygen species

**Figure 1:**
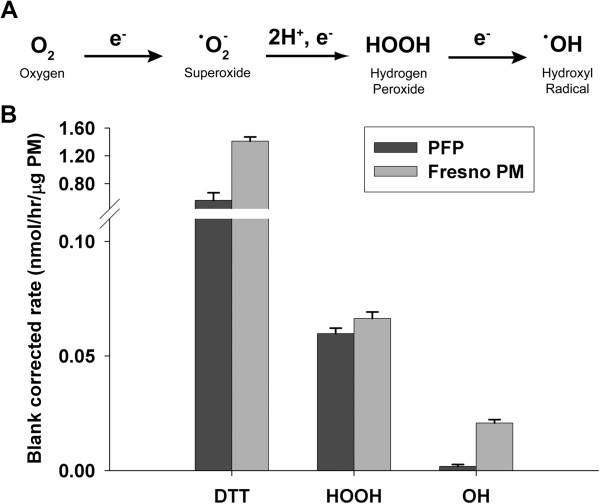
**PFPs generate reactive oxygen species. (A)** Chemical reaction showing the multistep reduction of molecular oxygen into reactive oxygen species. **(B)** Dithiothreitol consumption (n=10), and the generation of hydrogen peroxide (n=3), and hydroxyl radical (n=5) were assayed for PFP and compared to urban ambient PM_2.5_ collected from Fresno, CA.

As shown in Figure 1A, molecular oxygen is a viable electron acceptor that generates ROS (e.g. superoxide, hydrogen peroxide and hydroxyl radicals), both through cellular respiration and production by PM [24,25]. To define the metals concentration in PFP, which can function as a source of ROS in addition to PAHS, we quantified 8 transition metals in collected PFP. The concentrations of transition metals in PFP were all below the limit of quantification, with the exception of zinc. In contrast, metal concentrations are much higher in a typical ambient PM_1.8_ sample from Fresno, California (Table 1). PAH concentrations were also compared using historical data [21,26]. As shown in Table 2, PAH species were present in sub-nanogram per cubic meter concentrations in both PFP and ambient PM_1.8_, but distribution of PAHs differed between the two particle types. Higher concentrations of benz(a)anthracene and pyrene species were detected in PFP, but substituted fluoranthene and perylene species were undetectable, as compared with ambient PM_1.8_. To quantify the oxidative potential of PFPs, we measured dithiothreitol (DTT) consumption as well as the generation of hydrogen peroxide (HOOH) and hydroxyl radical (·OH) in a surrogate lung fluid. As a comparison, we also measured the oxidative potential of the same Fresno PM_1.8_ sample listed in Table 1. Since the oxidative potential of ambient PM differs from sample to sample, this Fresno sample is simply an example and not an average ambient response. Figure 1B illustrates that PFPs can generate ROS: they can both oxidize DTT and produce HOOH. PFPs do not generate ·OH; this is consistent with their very low transition metal concentrations, since metals are thought to be the dominant pathway for converting HOOH into ·OH. In contrast, the typical Fresno PM sample does produce ·OH (Figure 1B); likely because of its high levels of soluble iron and copper (Table 1). However, PFPs and Fresno PM have a similar response in the DTT and HOOH assays, indicating that PFPs can produce similar oxidative potential as ambient PM.

**Table 1:** **Soluble metals analysis on PM filters**^**a**^

**Metal**	**PFP soluble metals (μg metal/g PM)**	**Fresno soluble metals (μg metal/g PM)b**
Co	< 3.2	n/a
Cr	< 0.84	n/a
Cu	< 1.0	344 ± 137
Fe	< 25	262 ± 32
Mn	< 1.8	397 ± 56
Ni	< 3.4	n/a
V	< 0.68	17 ± 3
Zn	7.8 ± 4.9	n/a

n/a indicates data that are not available. Measurements are the average ± standard deviation.

^a^) Filter blank corrected soluble concentrations of 8 common transition metals were measured in PFP samples, an urban PM_2.5_ sample from Fresno, CA. Only zinc (Zn) was detected above background levels in the PFP samples.

^b^) from Shen et al., [30].

^c^) "< value" indicates a result less than the limit of quantification (10 × SD of the blank).

**Table 2:** Comparison of selected PAH concentrations between PFP and Fresno ambient PM1.8

	**PFP (Particulate Phase)a ng/m3**	**Fresno ambient PM1.8b ng/m3**
Benz(a)anthracene	0.08	0.01
Benzo(b)fluoranthene	n.d.c	0.05
Benzo(e)pyrene	n.d.	0.03
Benzo(GHI)fluoranthene	n.d.	0.02
Benzo(GHI)perylene	n.d.	0.04
Benzo(k)fluoranthene	n.d.	0.02
Chrysene	0.08	0.04
Coronene	0.08	0.03
Fluoranthene	0.08	0.02
Pyrene	0.15	0.03

a adapted from Chan et al. [21].

b adapted from Tablin et al. [26].

c denotes not detected.

### PFP induces cellular oxidative stress

**Figure 2:**
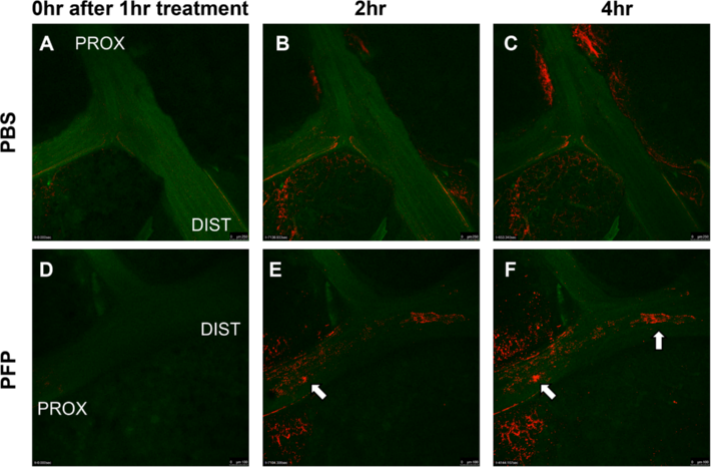
***Ex vivo *oxidative stress detection.** Rat right cranial lobes were dissected to expose the main axial airway and airways were treated with either PFP or PBS. Lungs were incubated with CellROX, a fluorescent dye that indicates oxidative stress, and washed with Live Cell Imaging solution after 1 hour of exposure and imaged continuously. Representative pictures from lungs taken directly **(A, D)**, 2 hours **(B**, and **E)** and 4 hours **(C** and **F)** after a 1 hour treatment of either PBS **(A-C)** or PFP **(D-F)**. Substantially more CellROX fluorescence could be observed in the airway lumen of the PFP-treated lung compared to PBS controls in a time dependent and proximal (PROX) to distal (DIST) manner. Focal patches of cells with oxidative stress were observed (arrows) increased over time in the PFP-treated lungs.

Next, we visualized the production of oxidative stress in the conducting airway epithelium *in situ* by instilling PFPs and CellROX Deep Red fluorescent oxidative detector in an *ex-vivo* model that preserves the 3-dimensional architecture and normal cell populations of the conduction airways. CellROX, a cell-permeable probe that fluoresces upon oxidation by ROS was used as a marker of oxidative stress. Figure 2 shows a time-course experiment comparing 1 hour of treatment of either PFP or PBS (vehicle control) instilled directly into rat bronchi in a proximal to distal direction. CellROX staining, indicative of oxidative stress, was not observed in either PBS or PFP instilled airways immediately after 1 hour of treatment (Figure 2A, D). Bronchiolar epithelial oxidant stress was absent in PBS controls. However, we observed mild parenchymal staining, possibly due to trauma during the dissection preparation process or from cellular instability during continuous imaging. Nevertheless, 2 hours following the 1 hour PFP treatment, CellROX fluorescence was seen in the cells linings the lumen of the treated bronchi (Figure 2E). This progressed over time in a proximal to distal manner (Figure 2F). This can be seen more clearly in the time-course videos, which are presented in the Supplement: PBS control (Additional file 1: Video S1), PFP treated (Additional file 2: Video S2).

**Figure 3:**
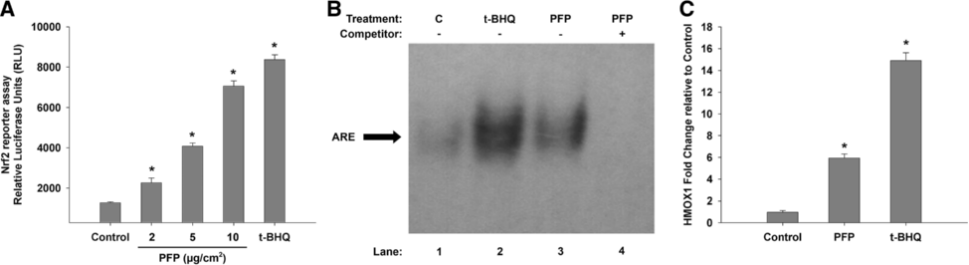
***In vitro *Nrf2 reporter assays.** Human U937 macrophages were transiently transfected with the Nrf2 luciferase reporter construct and subsequently treated with PFP or t-BHQ (positive control). **(A)** A dose-dependent increase in Nrf2 activity was observed after PFP treatment. Treatment with 2 μg/cm^2^ PFP yielded a significant response compared to controls. At 10 μg/cm^2^, the Nrf2 reporter generated similar levels compared to t-BHQ. **(B)** PFP treatment increases binding activity of Nrf2 at a consensus ARE binding element using GMSA with ARE consensus oligonucleotide after treatment of U937 macrophages with PFP (2 μg/cm^2^) and t-BHQ (10 μM) for 90 minutes. **(C)** HMOX1 mRNA was upregulated approximately 6-fold after 2 μg/cm^2^ PFP treatment compared to controls. Data are presented as mean+SEM (n=3 separate experiments), * significantly different compared to control.

We have previously shown that gene expression of both non-enzymatic and enzymatic antioxidants, especially those that utilize glutathione, are differentially altered 24 hours after PFP exposure depending on the post-exposure time and age [21]. We hypothesized that exposure to PFP activates the oxidative stress sensitive transcription factor Nrf2 and upregulates phase II detoxification genes containing the antioxidant response element (ARE). To test this hypothesis, we measured Nrf2 activation with an *in vitro* luciferase reporter assay. As shown in Figure 3A, Nrf2 activation increased in a dose-dependent manner with PFP treatment. Even at the lowest dose of 2 μg/cm^2^, Nrf2 activity was significantly upregulated 2-fold above controls (*p<0.05*). At 10 μg/cm^2^, Nrf2 activity was upregulated almost 6-fold above controls (*p*<0.01), at levels comparable to 10 μM of tert-butylhydroquinone (t-BHQ), a known Nrf2 inducer. No cytotoxic effects were observed at the highest concentration of 10 μg/cm^2^ PFP (data not shown).

EMSA studies (Figure 3B) confirmed that the increased Nrf2 activation is associated with an increased binding activity of the ARE consensus element which regulates the expression of HMOX1 and NQO1 for instance [27]. Next, we measured mRNA levels of an oxidative stress marker, HMOX1. The promoter region of HMOX1 contains ARE and has been shown to be Nrf2 inducible, and well correlated with both DTT consumption and PAH content in ultrafine PM [14,16]. We treated cells at 2 μg/cm^2^, the lowest dose from our dose-dependent data and observed a nearly 6-fold HMOX1 induction (*p<0.01*) after 4 hours of treatment. Treatment with Nrf2 activator t-BHQ increases HMOX1 levels; nearly 15 fold greater than controls (Figure 3C).

### PFP variably upregulates antioxidants in vivo

To measure antioxidant enzymes related to these in vitro studies, we used an in vivo exposure followed by an acute time course. We measured total antioxidant capacity in the lung tissue, along with mRNA and protein expression of 3 categories of antioxidants: oxidative stress (HMOX1), superoxide metabolism (SOD1, SOD2), and hydrogen peroxide sensors (CAT, PRDX6).

**Figure 4:**
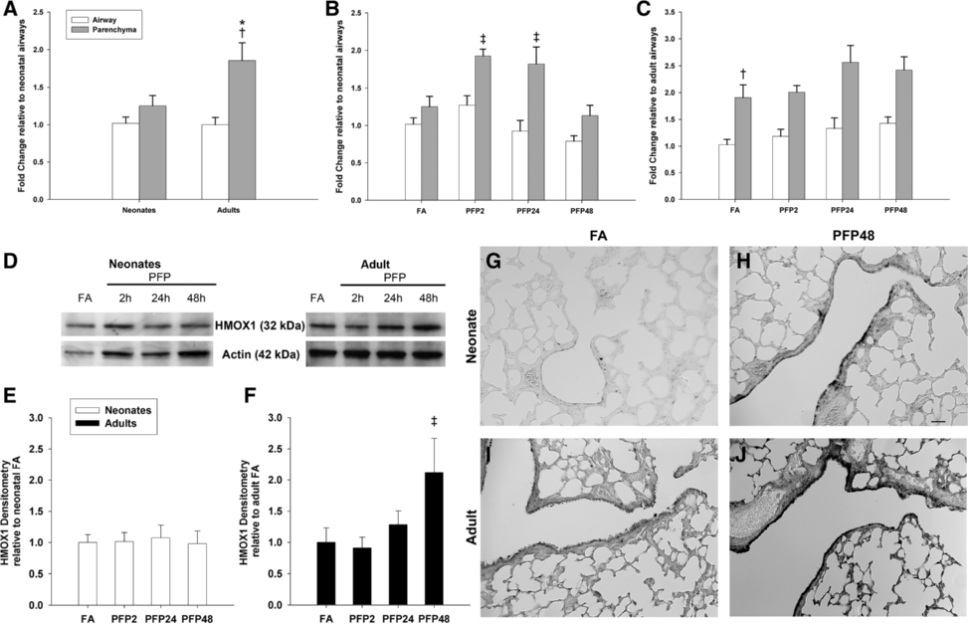
**HMOX1 mRNA and protein expression.** Animals were whole body exposed to FA or PFP for 6 hours, and sampled at 2, 24 and 48 hours post exposure; denoted as PFP2, PFP24 and PFP48. RT-PCR expression in airway and parenchymal compartments in neonatal and adult rats exposed to PFPs. **(A)** HMOX1 expression between compartments was similar in neonatal FA controls, but was greatest in adult parenchyma compared to both adult airways and neonatal parenchyma. **(B)** After PFP exposure, HMOX was transiently upregulated at PFP2 and PFP24 in the neonatal parenchyma. **(C)** Compared to neonates, adults had a delayed upregulated HMOX1 expression in the parenchyma that persisted to PFP48. Data are presented as mean+SEM (n=5-7 rats/group, in each compartment), * significantly different compared to neonates in the same compartment, † significantly different compared to airways in the same age, ‡ significantly different compared to FA in the same compartment. **(D)** Representative HMOX1 Western blot with actin loading control. **(E)** HMOX1 blots were quantified and revealed no exposure dependent effects in the neonatal lung. **(F)** HMOX1 protein expression trended upwards at 24 hours and reached statistical significance at PFP48. Data is presented as mean+SEM (n=6 rats/group) ‡ significantly different compared to FA in the same age. HMOX1 immunohistochemical detection of protein expression (n=6 rats/group) is presented in neonatal **(G-H)** and adult **(I-J)** of FA controls **(G, I)** and PFP48 **(H** and **J)** groups. HMOX1 protein was significantly enhanced in neonatal airways in the PFP48 group compared to FA. Additionally, robustly expressed HMOX1 protein was increased in both adult airways and parenchyma at PFP48 exposure. Scale bars are 50 μm.

First, we measured the total antioxidant capacity (TAC) of whole lung homogenate of adult and neonates reared in either filtered air (FA) or exposed to PFPs and allowed to recover for 2, 24 and 48 hours post exposure (PFP2, 24, 48). TAC measures were similar among neonates and adults, and no age or exposure effects were observed (Table 3). This was a surprising result, so we estimated airway and alveolar deposition following PFP exposure, and calculated deposition fractions of 0.195 and 0.15 for the adult and neonatal lung, respectively, agreeing with published deposition of 0.2 [28]. Using average body weights of 16g for neonates and 300g for adults, minute ventilation (MV) were calculated to be 25 ml/min in neonates, and 384 ml/min in adults. The estimated deposited dose (DD) was determined, where DD_neonate_ = 1.89 ng/g and DD_adult_ = 2.01 ng/g. Since the DD were similar between the two ages, we used lung compartment specific approaches and analyzed selected antioxidant enzymes. To determine whether exposure causes oxidative stress, we measured a typical marker, HMOX1. As shown in Figure 4, basal HMOX1 mRNA levels were comparable in between lung compartments in neonates. Conversely, HMOX1 was maximally expressed in the adult parenchyma while HMOX1 airway expression was similar to neonates. Post PFP exposure, we observed transient upregulation in the neonatal parenchyma; HMOX1 mRNA were significantly upregulated between PFP2 (*p*<0.01) and PFP24 (*p*<0.05) groups. Conversely, adult HMOX1 expression was largely unchanged. HMOX1 protein quantification revealed no exposure effects in neonates. On the contrary, adult HMOX1 was significantly upregulated at PFP48 (*p*<0.05). Spatial localization of HMOX1 revealed higher basal HMOX1 levels in adults. Post PFP exposure, neonatal HMOX1 protein was present diffusely in the parenchyma and densely localized in patches of bronchiolar epithelium. Contrastingly, adult HMOX1 protein was abundant in both the parenchyma and the bronchiolar epithelium.

**Table 3:** Total antioxidant capacity assay

	**FA**	**PFP2**	**PFP24**	**PFP48**
**Adults**	103±6	97±9	115±10	101±5
**Neonates**	109±19	102±10	86±18	109±14

### PFPs alter specific antioxidant enzymes in an age and time-dependent manner

**Figure 5:**
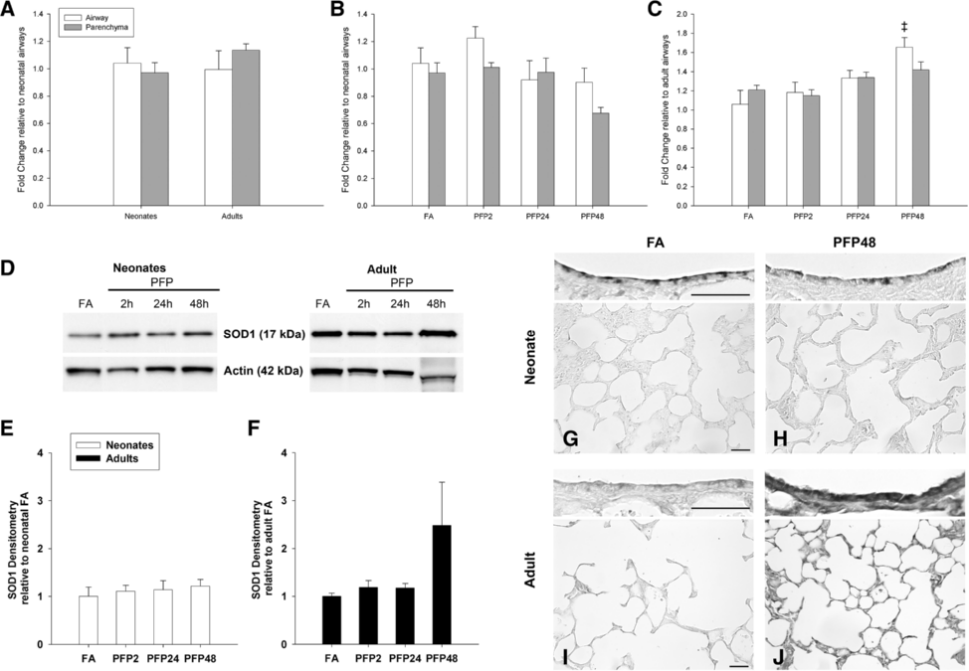
**SOD1 mRNA and protein expression.** RT-PCR: **(A)** SOD1 mRNA was similar in FA controls between both lung compartment and age. **(B)** Neonates had decreased SOD1 mRNA expression in PFP48. **(C)** Adults had increased airway SOD1 mRNA at PFP48. Data are presented as mean+SEM (n=5-7 rats/group, in each compartment), ‡ significantly different compared to FA in the same compartment. Western blotting: **(D)** Representative SOD1 blot with actin. **(E)** Neonatal whole lung SOD1 protein expression was unchanged post exposure. **(F)** Adult whole lung SOD1 trended upwards at PFP48, but was statistically insignificant. Data are presented as mean+SEM (n=6 rats/group). **(G-J)** SOD1immunohistochemical detection of SOD1 protein (n=6 rats/group) in neonatal lung tissue was unchanged post exposure. Contrastingly, intense airway and parenchymal SOD1 protein expression was observed in adult PFP48. Scale bars are 50 μm.

**Figure 6:**
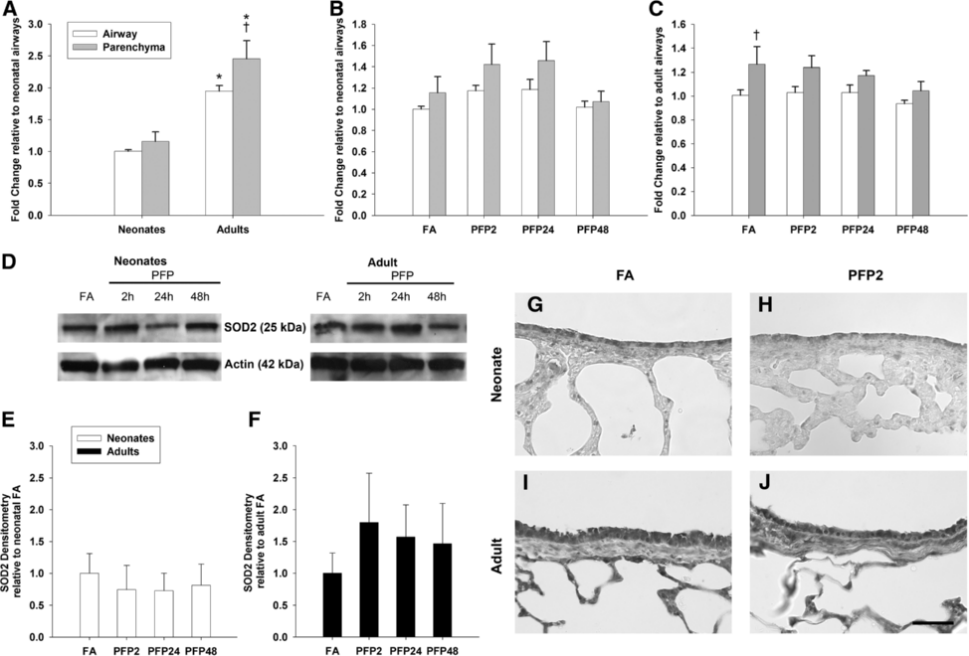
**SOD2 mRNA and protein expression.** RT-PCR: **(A)** SOD2 mRNA expression is higher in adults, and highest in the adult parenchyma. **(B)** No exposure effects on SOD2 mRNA were observed in neonates. **(C)** Adult SOD2 mRNA was decreased in PFP48. Data are presented as mean+SEM (n=5-7 rats/group, in each compartment), * significantly different compared to neonates in the same compartment, † significantly different compared to airways in the same age, ‡ significantly different compared to FA in the same compartment. Western blotting: **(D)** Scan of representative SOD2 and actin blots. **(E)** Neonatal whole lung SOD2 protein expression was unchanged with exposure, and **(F)** adult whole lung SOD2 protein trended upwards at PFP2, but was statistically insignificant. **(G-J)** Immunohistochemical localization of SOD2 in lung (n=6 rats/group). SOD2 protein was more abundant in adults compared to neonates, but no exposure specific differences were observed. Scale bar is 50 μm.

After establishing the incidence of oxidant stress, we examined the production of superoxide, the initial ROS species formed in the sequential reduction of molecular oxygen (Figure 1A). There are two main enzymes that perform cellular superoxide metabolism: cytosolic copper-zinc superoxide dismutase (SOD1) and mitochondrial manganese superoxide dismutase (SOD2). We measured mRNA and protein of both SOD1 (Figure 5) and SOD2 (Figure 6) in lung tissue. Basal cytosolic SOD1 mRNA levels were similar between lung (parenchyma and airway) compartments and did not change with age. Conversely, SOD2 mRNA were significantly greater in adults in both lung compartments. After PFP exposure, no discernible differences were observed in the neonatal lung in either superoxide dismutase isoform. However, SOD1 mRNA was significantly elevated in the adult airways at PFP48 (*p<*0.01). We did not find any exposure-related changes in SOD1 or SOD2 protein expression in neonates, but found that SOD1 was upregulated more than 2-fold at PFP48, and that SOD2 was approximately 1.7-fold higher at PFP2 in PFP exposed adults. Immunohistochemistry revealed similar results; neonatal superoxide dismutase proteins remained unaffected post-exposure, but heavy SOD1 protein expression in both adult airway and parenchymal tissue were noted at PFP48.

**Figure 7:**
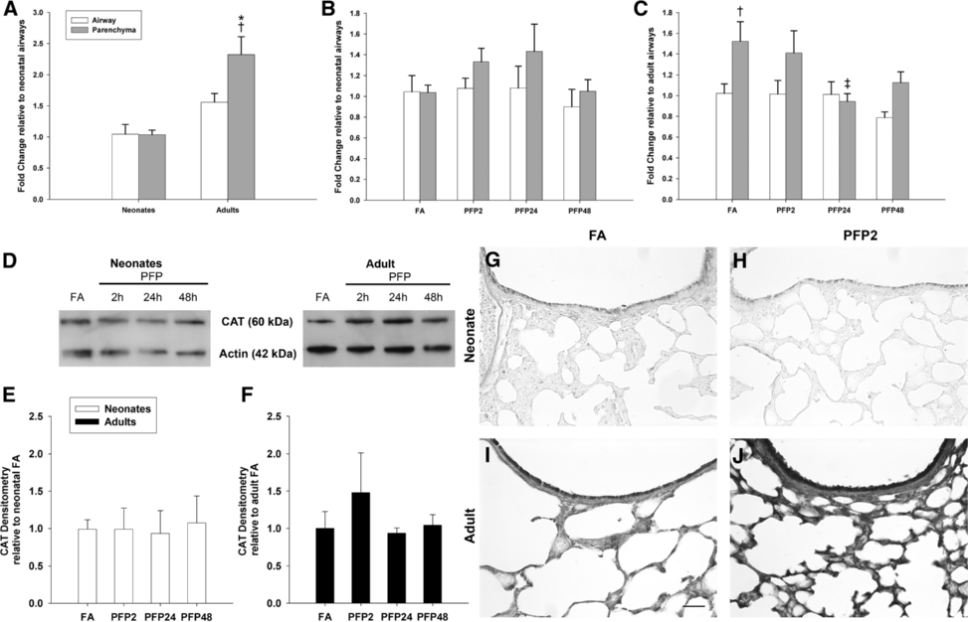
**CAT mRNA and protein expression. RT-PCR: (A) CAT mRNA was most abundant in the adult parenchyma, compared to neonates and the airway compartment. (B)** Neonatal CAT mRNA is unaffected by PFP exposure. **(C)** Adult CAT expression was significantly decreased at PFP24 and PFP48. Data are presented as mean+SEM (n=5-7 rats/group, in each compartment), * significantly different compared to neonates in the same compartment, † significantly different compared to airways in the same age, ‡ significantly different compared to FA in the same compartment. **(D)** Representatively CAT and actin Western blots. **(E)** Neonatal CAT protein was unchanged post exposure. **(F)** Adult CAT protein expression trended upwards transiently 2 hours post exposure, but this interaction was insignificant. **(G-J)** Immunohistochemical localization of CAT protein (n=6 rats/group) in lung tissue: Under FA conditions, adults had more abundant CAT protein than neonates. Similar to western blotting, intense protein localization to both airway and parenchyma tissue was observed. Scale bar is 50 μm.

**Figure 8:**
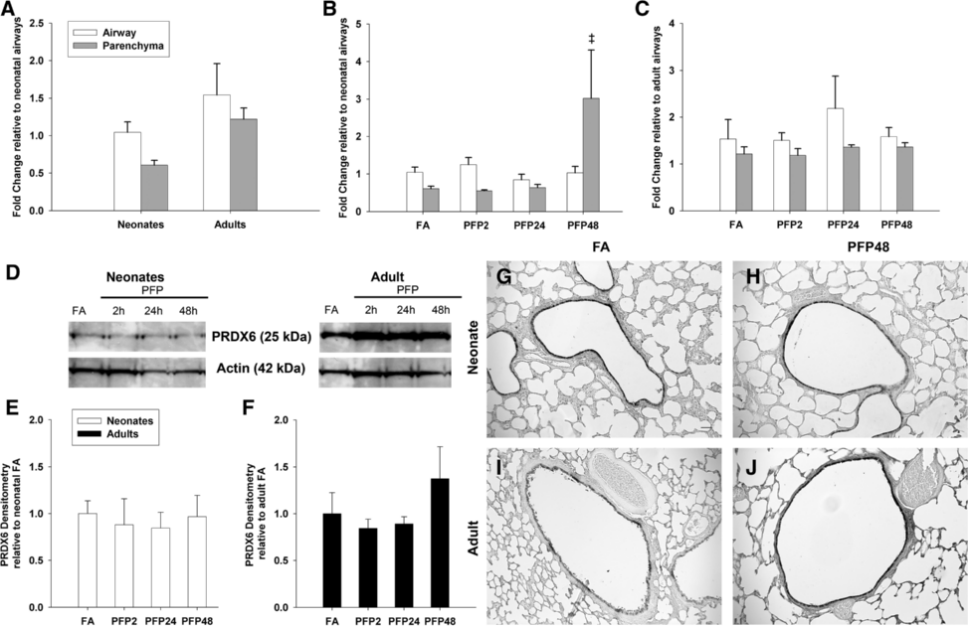
**PRDX6 mRNA and protein expression. RT-PCR: (A) Basal PRDX6 mRNA was similarly expressed between ages and compartments. (B)** Neonatal parenchymal PRDX6 mRNA was increased 48 hours post exposure. Data are presented as mean+SEM (n=5-7 rats/group, in each compartment), ‡ significantly different compared to FA in the same compartment.** (C)** Adult PRDX6 mRNA was unaffected by PFP. **(D)** Scan of PRDX6 and Actin Western blots. **(E)** Neonatal PRDX6 protein expression was unchanged by PFPs. **(F)** Adult PRDX6 protein expression trended upwards at PFP48. **(G-J)** Immunohistochemical localization of PRDX6 protein in lung tissue (n=6 rats/group). No substantial age and compartment differences were observed post exposure. Scale bar is 50 μm.

Finally, we measured the levels of two antioxidant enzymes responsible for the breakdown of hydrogen peroxide (HOOH) - catalase (CAT) and peroxiredoxin VI (PRDX6). As with the previous enzymes, we quantified mRNA and protein and used immunohistochemistry to visualize these two antioxidant enzymes within the lung. Figure 7 shows results from the studies of CAT, which catalyzes the dismutation of 2 molecules of HOOH into H_2_O and molecular oxygen. Constitutive CAT levels revealed that adults have the highest expression levels, especially in the parenchyma. Although we were unable to observe exposure effects on neonatal CAT, a time-dependent decrease was seen in adult CAT mRNA in PFP24 (*p*<0.05) group. Protein quantification mirrored the lack of response in neonates, with CAT protein levels remaining steady post exposure. Interestingly, we observed a transient but insignificant upregulation in adult CAT levels in the PFP2 group. CAT protein levels reverted to FA levels at PFP24 and PFP48, corresponding with decreases seen in mRNA. CAT immunohistochemistry revealed higher expression in the adults. Neonatal CAT immunostaining was supportive of the protein quantification; we did not observe any changes post exposure. Similarly, adult CAT protein was abundant in both the airways and the parenchyma at PFP2, but reverted back to FA levels at subsequent recovery times (data not shown). Finally, we assessed mRNA and protein expression of PRDX6 (Figure 8). PRDX6 is a highly conserved peroxidase present most abundantly in the lung [29]. Basal PRDX6 mRNA levels were consistent across age and lung compartments. Post exposure, we observed a significant increase in neonatal parenchymal mRNA for PRDX6 (*p*<0.01) that was not replicated in adults. However, protein levels were inconsistent with mRNA data, and no statistically significant differences were seen in either neonates or adults. Immunohistochemical detection of PRDX6 protein revealed highly abundant levels in the bronchiolar epithelium in both ages, but similar to the protein quantification results, we did not observe any exposure-related differences in expression for either age. A summary of PFP induced changes in antioxidant expression is compared (Table 4).

**Table 4:** Summary of PFP induced antioxidant expression

	**Assay**	**Lung compartment**	**Neonates**	**Adults**
**HMOX1**	RT-PCR	Airway	NC	NC
		Parenchyma	+	+
	WB	Whole Lung	NC	+
	IHC	Airway	+	+
		Parenchyma	NC	+
**SOD1**	RT-PCR	Airway	NC	+
		Parenchyma	-	NC
	WB	Whole Lung	NC	+
	IHC	Airway	NC	+
		Parenchyma	NC	+
**SOD2**	RT-PCR	Airway	NC	NC
		Parenchyma	NC	-
	WB	Whole Lung	NC	NC
	IHC	Airway	NC	NC
		Parenchyma	NC	NC
**CAT**	RT-PCR	Airway	NC	NC
		Parenchyma	NC	-
	WB	Whole Lung	NC	NC
	IHC	Airway	NC	+
		Parenchyma	NC	+
**PRDX6**	RT-PCR	Airway	NC	NC
		Parenchyma	+	NC
	WB	Whole Lung	NC	NC
	IHC	Airway	NC	NC
		Parenchyma	NC	NC

## Discussion

We accomplished three goals in this study. First, we determined the extent of ROS generation by PFPs. Second, we examined whether ROS in PFPs were sufficient to cause oxidative stress and activate the Nrf2 antioxidant response pathway using an *ex vivo* airway epithelium model and in an *in vitro* reporter system. Third, we compared antioxidant responses by measuring expression of three antioxidant enzyme families in neonatal and young adult rat lungs. We generated PFP as a surrogate for diluted vehicular exhaust. Animals were whole-body chamber exposed to 22.4 ± 5.6 μg/m^3^ PFPs, where particle number was measured to be 9.4 ± 0.5 × 10^4^ particles/cm^3^, consistent with ultrafine particle values reported 30 meters downwind from a major Los Angeles, CA highway (approx. 5.0 × 10^4^ particles/cm^3^; 65nm particles) [30]. The PFP environment is rich is organic compounds, with 2-methylnaphthalene (35.9 ng/m^3^) and naphthalene (15.4 ng/m^3^) as the most abundant PAH present in the vapor and particle phases, respectively [21]. Gasoline and diesel fuels have high concentrations of naphthalene, at 2600 mg/L and 1600 mg/L, respectively [31]. While a portion of PAHs in diesel exhaust emission is formed during fuel combustion, the majority of PAH are thought to be fuel-derived PAHs that are not destroyed during combustion. Measurements of vehicle exhaust from catalyst-equipped gasoline-powered motor vehicles emit approximately 1000 μg/km emissions of 2-methylnaphthalene and naphthalene, respectively [32]. Many PAHs present in PM have been shown to be capable of redox cycling and are carcinogenic [33-35]. The current study shows that, at environmentally relevant levels, PFP elicits significant oxidant effects, capable of activating Nrf2 and antioxidant enzymes.

PFPs are potent ROS generators that are capable of generating lung oxidative stress and activating the Nrf2-ARE pathway using *in vitro* and *ex vivo* models. We chose the DTT, HOOH and ·OH assays to measure the ability of PM to produce ROS through reduction of molecular oxygen. Both DTT loss and HOOH production can be from transition metals and quinones, while ·OH production typically requires transition metals [14,25]. In the current study, the PFP DTT response of 0.56 nmol/hr/μg PM is well within the typical range of ambient PM reported in the literature [36]. PFPs are rich in PAHs [21], which can be converted to redox-active quinones *in vivo*. Further, molecular oxygen is a viable electron acceptor that generates ROS (e.g. superoxide, hydrogen and hydroxyl radicals), both through cellular respiration and production by PM [24,25]. We hypothesized that through these mechanisms, PAHs indirectly play the primary role in ROS generation. Transition metals are another major source of ROS from PM [37,38], but we expected low concentrations of metals in our PM samples. Measurements of 8 common transition metals from PFPs found only zinc (Zn) above the detection limit. Zinc cannot redox cycle in a cell-free system and is not active in either the DTT or ·OH assay, so it is unlikely to produce any of the chemically generated ROS observed here [36,39]. Thus, organic species in the PFP are likely responsible for DTT loss and HOOH generation in our chemical assays.

ROS from PM exposures have been implicated in causing oxidative stress [13,40]. To visualize and detect oxidative stress in the 3-dimensional lung environment, we used a cell-permeable probe (CellROX) that fluoresces upon oxidation by ROS in a novel *ex vivo* model. We unequivocally showed that PFPs cause oxidative stress in a proximal to distal manner in the bronchiolar epithelium; the same direction that particles were instilled. Furthermore, as seen in the supplemental video, PFP treatment causes minor sloughing of CellROX positive cells in the bronchiolar epithelium.

We used the human macrophage cell line (U937) as an *in vitro* model to measure Nrf2 activation upon PFP exposure. Macrophages are one of the major alveolar cell types in the alveolar wall that play a critical role in homeostasis, host defense and response to foreign substances. Further, alveolar macrophages are responsive to organic and inorganic pollutants, such as diesel particulate and urban dust stimuli [41], promoting the release of proinflammatory cytokines and chemokines and increased activity of antioxidant enzymes. Nrf2-ARE is a key cellular defense pathway against ROS and oxidative stress. Under normal conditions, Nrf2 is cytosolic and constitutively bound to repressor Keap1. Nrf2 has a short half-life of ~15 minutes and is rapidly degraded by ubiquitinated proteosomic degradation [42]. ROS disrupts Keap1-Nrf2 binding, where Nrf2 is subsequently translocated to the nucleus, is phosphorylated and complexes with small Maf proteins to induce transcription of ARE-responsive phase II detoxification genes [43,44]. The Nrf2-luciferase reporter assay and EMSA demonstrates a clear dose-dependent increase in Nrf2 activity and increased ARE binding activity after PFP treatment. We confirmed Nrf2 induction through mRNA induction of a typical ROS and oxidative stress responsive ARE-containing enzyme, HMOX1. With these data, we unequivocally showed that PFP treatment is capable of activating Nrf2-ARE *in vitro*.

To verify these results in an *in vivo* system, 7-day old neonates and young adult rats were exposed to 6 hours of PFPs, and lung tissue was assessed at 2, 24 and 48 post exposure (designated PFP2, PFP24, and PFP48). Compared with humans, the neonatal rats used in this study are equivalent to newborn infants, while 8 week old rats are similar to teenagers [45]. This acute exposure tests the two extremes of a critical developmental and growth window for the lung. A limitation of our study is the increased number of confounding factors, especially prevalent in developmental studies. In the *in vivo* inhalation studies, neonatal animals were exposed to PFPs along with the dams to reduce weaning stress, along with providing a source of warmth and nutrition. Since neonatal animals are known to group together closely with their dam, maternal filtering may affect the inhaled PFP dose. Furthermore, neonatal proximity with the dam and differential feeding patterns also increases the variation seen between animals. These factors may have an undetermined effect on the deposited dose, and could possibly affect deposited dose estimations. Due to the heterogeneous nature of the lung, and the fact that distinct cell populations exist in separate lung compartments that may respond to PFP differently, we microdissected the lung tissues when practicable and used site-specific approaches to measure expression patterns of antioxidant enzymes. We first measured HMOX1, which is pervasive in the lung [14]. After a single PFP exposure, we saw significant HMOX1 parenchymal mRNA upregulation in both neonates and adults. Surprisingly, and unlike adult animals, mRNA upregulation did not translate to quantifiable HMOX1 increases in the whole lung homogenate nor in the parenchyma. The failure of neonates to sufficiently upregulate HMOX1 in the parenchyma has multiple implications for the developing lung. It is estimated that approximately 50 million alveoli are present at birth, which geometrically divides to over 300 million upon reaching adulthood [46]. Unnecessary cell turnover may disrupt alveolar maturation and investigators have previously found that mice lacking HMOX1 have reduced surfactant protein-B expression and are more susceptible to endotoxin [47]. The lack of HMOX1 upregulation may, in part, explain the enhanced parenchymal cytotoxicity [21] we see in neonatal rats exposed to PFP.

Superoxide dismutases are a family of enzymes that reduce O_2_^-^ anions into HOOH. There are two main intracellular isoforms; cytosolic copper-zinc superoxide dismutase (SOD1) and mitochondrial superoxide dismutase (SOD2). Only SOD1 was upregulated after PFP exposure, suggesting that O_2_^-^ is present in the cytosol. Our results are in agreement with Laing *et al.,*[48], showing significant SOD1 but not SOD2 upregulation in mice exposed to concentrated ambient particles in Ohio. Furthermore, we found substantial SOD1 upregulation in the adult parenchyma after exposure, consistent with literature indicating that alveolar type II cells are more protected from oxidative stress due to higher expression of SOD1 and SOD2 [49]. Our results differ from previous animal models where oxidative stress was induced using hyperoxia and SOD1 upregulation was observed in both neonates and adults [50]. This lack of concordance suggests that hyperoxia and PM exposures may increase SOD1 expression by different mechanisms, especially in neonates. Although SOD has been shown to be present in 7-day old neonates [51], neither SOD isoform was upregulated in the neonate animals exposed to PFP. This reinforces our hypothesis that neonatal animals are uniquely susceptible to PFP due to their inability to respond and upregulate antioxidant enzymes, possibly due to overriding developmental programming that is in play during this period of active lung growth or because of the differentiation state of the affected cell populations.

CAT and PRDX6 are intracellular peroxidases present in peroxisomes and cytosol, respectively. Peroxidases enzymatically dismute 2 molecules of HOOH into water and molecular oxygen. CAT has high catalytic activity that is rate-limited only by the concentration of HOOH. Oxidative DNA damage has been shown to cause cell cytotoxicity, and ROS from PM_2.5_ has been implicated to cause DNA damage [33,52]. *In vitro* treatment with PM_2.5_ supplemented with exogenous CAT or SOD ameliorated oxidative DNA damage [53]. We clearly saw enhanced CAT immunostaining in adult animals in both lung compartments 2 hours post exposure, but neonatal CAT expression appeared to be less abundant than adults and expression was unchanged by PFP exposure. The lower expression and lack of CAT upregulation may have implications on neonatal susceptibility to air pollution as functional CAT is thought to have a protective role. Mice lacking CAT develop normally, but they remove HOOH at a reduced rate and are more susceptible to oxidative stress [54]. Epidemiological studies have found that a functional single nucleotide polymorphism in the promoter region of CAT, G-330A that results in higher blood CAT levels, is associated with lower risk of respiratory-related school absences in children [55] and a decreased risk of developing asthma [56].

PRDX6 is capable of reducing both hydrogen peroxide and phospholipid peroxides. However, it is dependent on the formation of glutathione S-transferase P (GSTP1) and PRDX6 heterodimer in the presence of glutathione (GSH) to generate peroxidase activity [57]. Although we saw increases in PRDX6 mRNA levels in neonatal parenchyma at 48 hours after PFP exposure, this was not translated into increased protein. However, it may not be surprising that we didn’t see any induction of PRDX6, because it may be unnecessary. PRDX6 has a turnover rate of over 5 μmol/min/mg protein [57], vastly exceeding the 0.07 nmol/hr/μg PM HOOH present in PFP. Alternatively, PRDX6 induction could be limited by GSH or possibly GSTP1. As we have previously shown, PFP exposure significantly diminishes the amount of reduced GSH within both the airways and parenchyma in neonates, but not in adults [22]. Additionally, unlike adults, neonates did not upregulate GSTP1 expression in response to PFP exposure. The inability to regulate either mRNA and/or protein expression of both PRDX6 and GSTP1, in combination with diminished glutathione in key target regions may play a role in neonatal susceptibility to PFPs.

In summary, the present study establishes that laboratory generated PFP is a potent generator of ROS. We further determined that PFP induces oxidative stress in both an *ex vivo* and *in vivo* models. Because our PM is low in metals, we infer that it is likely that the organic content of PM in an exhaust atmosphere is an important contributor to pulmonary susceptibility, particularly in the developing lung. Antioxidant enzyme expression was variably altered after PFP exposure, depending on age, lung compartment, and post-exposure recovery time underscoring the importance of site specific approaches to analyze biological effects of inhaled compounds. Cell populations are location-specific and have unique functions within the lung. The inability of neonates to upregulate antioxidants in the parenchyma provides a mechanism for the enhanced parenchymal toxicity to PFP we’ve previously reported [21-23]. From the data presented, we conclude that the HMOX1 and SOD1 mRNA and protein upregulation are suitable oxidative stress detection markers to measure effects of PFP exposure. Moreover, we posit that the enhanced susceptibility of neonatal mice to inhaled PFPs might be due to their inability to upregulate key oxidative stress response and antioxidant proteins that are needed to return to cellular homeostasis.

## Methods

### Particle generation

Premixed flame particle (PFP) aerosols were generated using a coannular premixed flame burner as detailed previously [20,21]. Briefly, a Pyrex-tube enclosed burner was fed a metered mixture of ethylene (212.4 cc/min), oxygen (289.2 cc/min) and argon (1499 cc/min) to generate the flame. A small flow of oxygen (52 cc/min) flowed through the outer annulus to stabilize the flame. Dry filtered air (FA) was added to the flow downstream and burner effluent passed through a heated 3-way catalyst to remove NO_x_ and CO. Finally, PFPs were diluted and mixed with clean HEPA and CBR (chemical/biological/radiological) FA prior to entering the inhalation exposure chamber. Chambers were maintained at −0.3 inches of H_2_O gauge pressure and temperature were maintained between 22.2 and 24.4°C. PFP are on average 70.6 ± 1.5 nm (geometric mean ± SD) as determined by Scanning Mobility Particle Sizer (SMPS) measurements. Particle mass concentration in the chamber was 22.4 ± 5.6 μg/m^3^ PFP (mean ± SD), and the total particle numbers was (9.37 ± 0.48) ×10^4^ (mean ± SD) determined used a condensation particle counter. Particles were high in organic carbon and had an EC:OC ratio of 0.58. Gas- and particle-phase concentrations of polycyclic aromatic hydrocarbons (PAHs) were 405 and 54 ng/m^3^, respectively.

### Surrogate lung fluid

The SLF used for *ex vivo* ·OH, HOOH, and soluble metals measurements was phosphate buffered saline (PBS) containing 10 mM phosphate buffer (7.2 mM sodium phosphate dibasic, 2.2 mM potassium phosphate monobasic) and 0.114M NaCl with a pH of 7.3-7.4 [39]. The PBS was treated with Chelex 100 (Bio-Rad, Hercules, CA) to remove trace metal contamination. On the day of the experiment, antioxidant stocks were freshly made and added to the PBS to make final concentrations of 0.20 mM ascorbate (Arcos Organics, Pittsburg, PA), 0.30 mM citrate (Fisher Scientific, Pittsburg, PA), 0.10 mM reduced glutathione (Sigma-Aldrich, St. Louis, MO) and 0.10 mM urate (Sigma-Aldrich). 10 mM of sodium benzoate (NF/FCC) was added to the SLF as a probe for ·OH measurements only.

### Ambient PM_1.8_ collection

Ambient PM_1.8_ was collected in Fresno, CA during the summer of 2008 from 10:00 am to 4:00 pm local time for two consecutive five-day periods with a two-day break, (August 24^th^ – 28^th^ and August 31^st^ – September 4^th^ 2008). PM_1.8_ were collected onto Teflon filters using a high volume sampler with a PM_1.8_ impaction stage. Additional PM collection details can be found in [38]. After collection, samples were stored at −20°C in the dark.

### PM incubation

A filter section with known PM mass (18.4 μg for PFP and 115.7 μg for ambient PM_1.8_) was incubated in the reaction mixture for soluble metals, DTT, ·OH and HOOH analysis along with 18.4 mM of trifluoroethanol as a filter wetting agent. Samples were stirred constantly on a shake table to extract PM. A blank filter of the same size was also analyzed and used to filter-blank correct all results.

### Hydrogen peroxide assay

The rate of formation of HOOH was quantified from PM in 4.0 mL SLF using the method previously described [38], except that we use the four antioxidants stated above instead of only ascorbate. Briefly, HOOH production from PM was measured in triplicate at 0, 0.5, 1 and 1.5 hours, and the rate of HOOH production was calculated from the slope of the linear response. The rate of HOOH production from the filter blank was subtracted from the rate of HOOH from PM. HOOH was quantified using HPLC with post-column derivatization and fluorescence detection (excitation 320 nm, emission 400 nm) [58]. Daily quality controls include a solution blank and positive control (250 nM copper) run with each experiment. HOOH calibrations were run daily, and the concentration of the HOOH stock was confirmed using UV–VIS absorption at 240 nm.

### Hydroxyl radical assay

The rate of ·OH formation from PM was quantified in 6.0 mL SLF with four antioxidants (see above) [39]. Briefly, ·OH was quantitatively trapped using a sodium benzoate probe. The stable product, p-hydroxybenzoic acid, was quantified using HPLC with UV–VIS detection using a standard p-hydroxybenzoic acid solid as a daily calibration. ·OH was measured in triplicate at 0, 1, 2, 4 and 24 hours and the rate of ·OH production was calculated from the slope of the linear data. The rate of ·OH production from a filter blank was subtracted from the rate of ·OH production from PM. A solution blank and a positive control (1.44 μM iron) were run daily for quality control.

### DTT assay

The DTT assay is a cell-free, *in-vitro*, chemical measure of the oxidative potential of PM which responds to trace redox-active chemicals in PM such as quinones and transition metals, and is described in detail in the literature [24,36]. Briefly, the loss of 100 μM of DTT (Arcos Organics) incubated with PM in 3.0 mL of 0.1 M phosphate buffer (pH 7.3-7.4) at 37°C is measured over time. The rate of DTT loss provides a quantitative measure of the oxidative potential of the PM under conditions of the DTT assay.

### Soluble metals analysis

A filter section (see above) was added to 0.50 mL of HOOH SLF and the solution was mixed on a shake table for 24 hours at room temperature. 0.40 mL of solution was filtered through a 0.22 μM PTFE filter (Millipore) into 3.6 mL of 3% nitric acid (trace metal grade). Soluble metals were quantified using an inductively coupled plasma mass spectrometer (ICP-MS) by the UC Davis interdisciplinary center for ICP-MS. A filter blank was also analyzed and the limit of quantification was calculated as 10 time the standard deviation of the blank [59].

### Cell culture and transient transfection

Human U937 monocytic cells were obtained from the American Tissue Culture Collection (Manassas, VA) and maintained in RPMI 1640 medium containing 10% fetal bovine serum (Gemini, Woodland, CA), 100 U penicillin, and 100 μg/ml streptomycin supplemented with 4.5 g/L glucose, and 1 mM sodium pyruvate. Cell culture was maintained at a cell concentration between 2 × 10^5^ and 2 × 10^6^ cells/ml. For differentiation into macrophages, U937 cells were treated with TPA (3 μg/ml) and allowed to adhere for 48 hr in a 5% CO_2_ tissue culture incubator at 37°C, after which they were fed with TPA-free medium. Differentiation state was assessed by attached cell phenotype and increased expression of MAC-2. U937 macrophages were treated in regular growth medium containing 10% FBS. PFPs were used at 2, 5 or 10 μg/cm^2^ growth area, equivalent to 10, 25 and 50 μg/mL. Concentrations of PFP are preferentially expressed in micrograms per square centimeter growth area because particles rapidly sediment onto the cell layer.

For transient transfection of U937 macrophages, the Nrf2 luciferase reporter construct (Promega, Madison, MI) was transfected via Nucleofector technology as described previously [60]. Briefly, 10^6^ U937 macrophages were resuspended in 100 μl Nucleofector Solution V (Amaxa GmbH, Köln, Germany) and nucleofected with 1.0 μg plasmid DNA using program V-001, which is preprogrammed into the Nucleofector device (Amaxa GmbH). Following nucleofection, the cells were immediately mixed with 500 μl of pre-warmed RPMI 1640 medium and transferred into six-well plates containing 1.5 ml RPMI 1640 medium per well. After 24 h transfection, macrophages were treated with PM or 10 μM t-BHQ (positive control) for 4 h. Luciferase activities were measured with the Luciferase Reporter Assay System (Promega) using a luminometer (Berthold Lumat LB 9501/16, Pittsburgh, PA). Relative light units are normalized to β-galactosidase activity and to protein concentration using Bradford dye assay (Bio-Rad, Hercules, CA).

### Cell viability assay

To assess the effect of PFP on viability of U937 macrophages, we used the trypan blue exclusion test. A 10 μl sample of re-suspended cell pellet was placed in 190 μl PBS with 200 μl trypan blue (0.5% dilution in 0.85% NaCl). After 5 minutes, 10 μl of the cell suspension was loaded into a hemocytometer and determined the proportion of nonviable to viable cells.

### Electrophoretic mobility shift assay (EMSA)

Nuclear extracts were isolated from U937 cells, as described previously [61]. In brief, 5 × 10^6^ cells were treated with PFP or t-BHQ for 90 min and harvested in Dulbecco’s PBS containing 1 mM PMSF and 0.05 μg/μl of aprotinin. After centrifugation, the cell pellets were gently resuspended in 1 ml of hypotonic buffer (20 mM HEPES, 20 mM NaF, 1 mM Na_3_VO_4_, 1 mM Na_4_P_2_O_7_, 1 mM EDTA, 1 mM EGTA, 0.5 mM PMSF, 0.13 μM okadaic acid, 1 mM dithiothreitol, pH 7.9, and 1 μg/ml each leupeptin, aprotinin, and pepstatin). The cells were allowed to swell on ice for 15 min and then homogenized by 25 strokes of a Dounce-homogenizer. After centrifugation for 1 min at 16,000 × *g*, nuclear pellets were resuspended in 300 μl ice-cold high-salt buffer (hypotonic buffer with 420 mM NaCl, and 20% glycerol). The samples were passed through a 21-gauge needle and stirred for 30 min at 4°C. The nuclear lysates were microcentrifuged at 16,000 × *g* for 20 min, aliquoted and stored at −80°C. DNA-protein binding reactions were carried out in a total volume of 15 μl containing 10 μg nuclear protein, 60,000 cpm of DNA ARE oligonucleotide (5’-agcacatgtgacatctctcctaag-3’), 25 mM Tris buffer (pH 7.5), 50 mM NaCl, 1 mM EDTA, 0.5 mM dithiothreitol, 5% glycerol, and 1 μg poly (dI-dC). The samples were incubated at room temperature for 20 min. Competition experiments were performed in the presence of a 100-fold molar excess of unlabeled DNA fragments. Protein-DNA complexes were resolved on a 4% nondenaturing polyacrylamide gel and visualized by exposure of the dehydrated gels to X-ray films. For quantitative analysis, respective bands were quantified using a ChemiImager™ 4400 (Alpha Innotech Corporation, San Leandro, CA).

### In vitro RNA isolation and real time reverse transcription-PCR

Total RNA was isolated from U937 cells using a Quick-RNA Mini prep isolation kit (Zymo Research, Irvine, CA), and cDNA synthesis was done as previously described [61]. Quantitative detection of heme oxygenase-1 (HMOX1, NCBI RefSeq: NM_010442.2) and Rps13 (NCBI RefSeq: NM_026533.3) genes was performed with a StepOnePlus™ Real-Time PCR System (Applied Biosystems) using the Fast SYBR Green Master Mix (Life Technologies, Grand Island, NY) according to the manufacturer’s instructions. DNA-free total RNA (1.0 μg) was reverse-transcribed using 4 U Omniscript reverse transcriptase (RT; Qiagen) and 1 μg oligo(dT)_15_ in a final volume of 40 μl. The primers for each gene were designed on the basis of the respective cDNA or mRNA sequences using OLIGO primer analysis software provided by Steve Rozen and the Whitehead Institute/MIT Center for Genome Research [62] so that the targets were 100–200 bp in length. Primers for human HMOX1 are: left primer ‘cacgcatatacccgctacct’ and right primer ‘ccagagtgttcattcgagca.’ Primers for human Rps13 are: left primer ‘gtccgaaagcaccttgagag’ and right primer ‘agcagaggctgtggatgact.’ PCR amplification was carried out in a total volume of 20 μl containing 2 μl cDNA, 10 μl 2 × Fast SYBR Green Master Mix, and 0.2 μM of each primer. The PCR cycling conditions were 95°C for 30 sec followed by 40 cycles of 94°C for 3 sec, and 60°C for 30 sec. Detection of the fluorescent product was performed at the end of the 72°C extension period. Negative controls were concomitantly run to confirm that the samples were not cross-contaminated. A sample with DNase- and RNase-free water instead of RNA was concomitantly examined for each of the reaction units described above. To confirm the amplification specificity, the PCR products were subjected to melting curve analysis.

### Ex-vivo based oxidative stress detection

Eight-week old male Sprague Dawley rats (Harlan Laboratories, Hayward, CA) were acclimated in CBR filtered air (FA) for a week prior to experimentation. Rats were provided with Laboratory Rodent Diet (Purina Mills, St. Louis, MO) and water *ad libitum*. Animals were euthanized by intraperitoneal (IP) injection of an overdose of pentobarbital (150 mg/kg). All animal experiments were performed as described in protocols approved by the University of California IACUC in accordance with guidelines set by the National Institute of Health. At necropsy, tracheas were cannulated, thorax opened and lung removed en bloc, inflated with low gelling temperature and processed as previously described [63]. The right cranial lobe was glued to a 22 mm^2^ coverslip (Corning Life Sciences, Lowell, MA) with Nexaband tissue adhesive (Abbott Animal Health, North Chicago, IL) and bisected in half to expose the main axial airway. Agarose was removed from the bronchiolar airways. Airways were directly treated with either 10 μg of PFP, dissolved in endotoxin free PBS (Enzo Life Sciences, Farmingdale, NY) at 5 μg/μl concentration or PBS only (vehicle control) by pipetting the substance onto the airway lumen in proximal to distal direction. The lungs were incubated in Live Cell Imaging Solution (Life Technologies, Grand Island, NY) at 37°C for 30 minutes. Then, 5 μM CellROX Deep Red Reagent (Life Technologies, Grand Island, NY) was applied directly onto the dissected lung and allowed to incubate for 30 minutes following manufacturer’s instructions. Lungs were washed 3 times with Live Cell Imaging Solution and imaged continuously on the Leica TCS LSI zoom confocal microscopes (Leica Microsystems, Wetzlar, Germany) using a 488 nm excitation laser. Experiments were repeated 5 times on separate days with different animals.

### Animal exposure protocol

Eight-week old male adult and newborn male Sprague Dawley rats with accompanying dams (Harlan Laboratories, Hayward, CA) were allowed to acclimate in FA until newborns reached 7 postnatal days of age in a 12 hour light/dark cycle starting at 7AM. Adult rats were housed in steel wire cages, while newborn rats and accompanying dams were housed in polycarbonate cages with wire tops and provided with Laboratory Rodent Diet (Purina Mills, St. Louis, MO) and water *ad libitum*. Six animals per group were exposed to 22.4 ± 5.6 μg/m^3^ PFP or FA atmosphere for 6 hours in two separate chambers as previously described [20,21]. Chambers are maintained at 22-23°C and 40-44% humidity. Animals were necropsied at 2, 24 and 48 hours following the single 6-hour exposure; treated groups will be designated as PFP2, PFP24 and PFP48, respectively. All animals were euthanized by an intraperitoneal injection of 0.5 ml/kg body weight pentobarbital and subsequently exanguinated prior to lung removal. At necropsy, the trachea was cannulated, thorax opened and lung removed en bloc for processing.

### Deposited dose estimation

PFP deposition in the adult and neonatal rat lungs were modeled and estimated. Adult deposition was modeled using Multiple Path Particle Dosimetry (MPPD) model v.2.1 (ARA, Albuquerque, NM) using PFP concentration at 22.4 μg/m^3^, or ng/L equivalents. Since the MPPD model does not calculate neonatal lung deposition, data for dose estimation were derived from 80nm particles as previously described [64]. Minute ventilation was calculated using the following formula: MW = 2.14 × BW^0.91^, where BW = body weight [64]. Using these values, the deposited dose (DD) was determined: DD (ng/g) = PFP concentration (ng/L) × MW (L/min) × deposition fraction × exposure duration (min) / BW (g).

### Total Antioxidant Capacity (TAC) Assay

Lungs were flash frozen in liquid nitrogen and stored at −80°C. Tissue was homogenized in cold PBS, centrifuged at 10,000 × g and supernatant collected for protein determination and subsequent TAC assay. Protein concentrations were determined with the Bradford assay (Bio-Rad, Hercules, CA). TAC was measured against uric acid standards using the OxiSelect Total Antioxidant Capacity Assay (Cell Biolabs, San Diego, CA) strictly following manufacturer directions. Values from uric acid equivalents were converted to copper reducing equivalents (CRE), where 1 mM uric acid = 2189 μM CRE. CRE sample values are proportional to the sample’s TAC.

### In vivo lung compartmental RNA isolation and RT-PCR

Lung compartmental RNA was isolated from microdissected intrapulmonary airways and surrounding parenchymal tissue from RNAlater (Ambion, Austin, TX) stabilized lung tissue using the Qiagen RNeasy Mini Kit (Qiagen, Valencia, CA) as previously described [65]. RNA purity was confirmed using spectrophotometric absorbance at 260/280 nm. Gene quantification in the airway and parenchymal compartments were performed using inventoried Taqman probes and primers (Applied Biosystems, Foster City, CA) listed in Table 5 as previously described [65,66]. Results were calculated using the comparative Ct method [67,68] using Hypoxanthine-guanine phosphoribosyltransferase (HPRT) as the reference gene. HPRT was chosen as the reference gene due to consistency by age and low variance between exposure groups as previously assessed [21,69]. Results are expressed as a fold change in gene expression relative to filtered air exposed animals of the same age, unless otherwise stated.

**Table 5:** Taqman gene expression assay catalog numbers

**Symbol**	**Assay ID**	**Gene name**	**NCBI RefSeq**
CAT	Rn00560930_m1	Catalase	NM_012520.1
HMOX1	Rn01536933_m1	Heme oxygenase (decycling) 1	NM_012580.2
PRDX6	Rn00821480_g1	Peroxiredoxin 6	NM_053576.2
SOD1	Rn00566938_m1	Superoxide dismutase 1, soluble	NM_017050.1
SOD2	Rn00690588_g1	Superoxide dismutase 2, mitochondrial	NM_017051.2
HPRT	Rn01527840_m1	Hypoxanthine-guanine phosphoribosyltransferase	NM_012583.2

### Western Blotting

Flash frozen lung tissue was homogenized in RIPA lysis buffer (Santa Cruz Biotechnology, Santa Cruz, CA) and protein concentrations determined with the Bradford assay (Bio-Rad, Hercules, CA). Samples were reduced for SDS-PAGE application, and 20–40 μg protein/lane was electrophoresed on 12% Bis-Tris polyacrylamide gels (Bio-Rad, Hercules, CA) and subsequently transferred onto a polyvinylidene fluoride (PVDF) membranes. The optimal amount of protein loaded was determined through a series of dilutions to optimize for quantification linearity. To reduce nonspecific antibody binding, membranes were incubated in 5% albumin and incubated with each of the following antibodies: rabbit anti-SOD1 (Abcam, Cambridge, MA) at 1:5000, rabbit anti-HMOX1 (Abcam) at 1:2000, rabbit anti-PRDX6 (LabFrontier, Seoul, Korea) at 1:4000, rabbit anti-catalase (Novus Biologicals, Littleton, CO) at 1:1000, rabbit anti-SOD2 (Novus Biologicals, Littleton CO) at 1:2000 and rabbit anti-actin (Abcam) at 1:1000 overnight. Protein bands were amplified using a horseradish peroxidase (HRP)-conjugated anti-rabbit secondary at 1:2500 (Abcam) and visualized using Amersham ECL reagents (GE Healthcare, Piscataway, NJ) on Amersham ECL Hyperfilm (GE Healthcare). Developed film was scanned on an Epson Precision 1640SU scanner (Epson America, Long Beach, CA) and protein bands quantified using ImageJ (NIH, Bethesda, MD). Results are expressed as relative abundance in protein expression relative to filtered air animals of the same age.

### Immunohistochemistry

Lungs were inflated with 37% formaldehyde vapor bubbled under 30 cm hydrostatic pressure for 1 hour as previously described [70,71]. Samples were stored in 1% paraformaldehyde for less than 24 hours prior to tissue processing and paraffin embedment. Paraffin sections on poly-L-lysine coated slides from all groups were stained simultaneously to minimized variability between runs and were immunostained for rabbit anti-SOD1 (Abcam, Cambridge, MA) at 1:3000, rabbit anti-HMOX1 (Abcam) at 1:250, rabbit anti-PRDX6 (LabFrontier, Seoul, Korea) at 1:3000, sheep anti-SOD2 (The Binding Site, San Diego, CA) at 1:2000 and sheep anti-CAT (The Binding Site) at 1:2000. The concentration of primary antibody was determined through a series of dilutions to optimize for staining density while minimizing background. This procedure was performed according to previously described methods [63,69] with several alterations. Following tissue hydration, endogenous peroxidase activity was quenched with a 10% solution of hydrogen peroxide. To eliminate nonspecific primary antibody binding, tissue sections were blocked with 5% albumin. Primary antibodies were allowed to incubate in a humidified chamber overnight at 4°C. Signal was amplified using the Vectastain IgG Avidin-Biotin-HRP Kit (Vector Labs, Burlingame, CA), and visualized using nickel chloride enhanced 3,3’-diaminobenzidine tetrachloride (Sigma-Aldrich, St. Louis, MO) as the chromagen. Controls included substitution of primary antibody with phosphate-buffered saline to ensure specific positive staining.

### Statistics

Data are reported as mean ± standard error of the mean (SEM) unless otherwise stated. Multivariate analysis of variance (MANOVA) was applied against age, compartment and exposure factors when appropriate. Pair-wise comparisons were performed individually using a one-way ANOVA followed by Dunnett’s post hoc tests against age and compartment controls using StatView (SAS, Cary, NC). *P* values of < 0.05 were considered statistically significant.

## Abbreviations

PM: Particulate matter; PAH: Polycyclic aromatic hydrocarbon; PFP: Premixed flame particles; FA: Filtered air; ROS: Reactive oxygen species; DTT: Dithiothreitol; HOOH: Hydrogen peroxide; ·OH: Hydroxyl radical; TAC: Total antioxidant capacity; SLF: Surrogate lung fluid; PBS: Phosphate buffered saline; HMOX1: Heme oxygenase 1; SOD1: Superoxide dismutase [Cu-Zn]; SOD2: Superoxide dismutase [Mn]; CAT: Catalase; PRDX6: Peroxiredoxin VI; ARE: Antioxidant response element; t-BHQ: Tert-butylhydroquinone; GSH: Glutathione (reduced); GSSG: Glutathione disulfide; GCL: Glutamate cysteine ligase; GPX: Glutathione peroxidase; GSR: Glutathione reductase; GST: Glutathione S-transferase; MW: Minute ventilation; BW: Body weight; DD: Deposited dose; MPPD: Multiple path particle dosimetry.

## Competing interests

One Author, Dr. Laura Van Winkle, has identified a potential apparent competing financial interest with the American Petroleum Institute (API). Dr. Van Winkle has received research grants from API to study the kinetics of naphthalene bioactivation and cytotoxicity in the respiratory system and has received honoraria from API for speaking at research conferences sponsored by API on naphthalene. API did not fund the work presented in the attached study and the research grant funded by API has complete freedom to publish the results regardless of whether they are in API’s interest, and without input from API, in keeping with University of California policy. The remaining authors declare they have no actual or potential competing financial interests.

## Authors’ contributions

JKWC, DSA and LSVW conceived and designed research; JKWC, JGC, SDK, CFV, SYK and DSA performed experiments; JKWC, JGC, CA and DSA analyzed data; JKWC, JGC, CFV, CA and LSVW interpreted results of experiments; JKWC and SDK prepared figures; JKWC and JGC drafted manuscript; JKWC, JGC, CFV, CA and LSVW edited and revised manuscript. All authors read and approved the final manuscript.

## Supplementary Material

Additional file 1: Video S1 Video of CellROX stained PBS control rat lung. Click here for file

Additional file 2: Video S2 Video of CellROX stained PFP treated rat lung. Click here for file
